# A High-Echoic Layer Surrounding the Heart Suggesting Cardiac Tamponade by Clotting

**DOI:** 10.1055/s-0041-1729912

**Published:** 2021-10-12

**Authors:** Ikuto Takeuchi, Jun Shitara, Youichi Yanagawa

**Affiliations:** 1 Department of Acute Critical Care Medicine, Juntendo Shizuoka Hospital, Izunokuni City, Shizuoka Prefecture, Japan

**Keywords:** aortic dissection, tamponade, Loeys–Dietz syndrome

## Abstract

A 16-year-old boy experienced a sudden loss of consciousness. On arrival, he was in cardiac arrest. An ultrasound study revealed a high-echoic layer surrounding the heart. He received a diagnosis of clotting cardiac tamponade. Urgent thoracotomy with pericardiotomy was performed, but he failed to obtain return of spontaneous circulation. Physicians should focus on not only low-echoic but also high-echoic areas to accurately diagnose clotting, which can result in a critical condition if not managed properly.

A 16-year-old boy experienced a sudden loss of consciousness at home in front of his parents. On arrival, he was in cardiac arrest with pulseless electrical activity. An ultrasound study revealed a high-echoic layer surrounding the heart with a high-echoic layer surrounding the ascending aorta as well ( Figs. 1 and 2 ). He received a diagnosis of clotting cardiac tamponade. Urgent thoracotomy with pericardiotomy was performed ( Fig. 3 ), but he failed to obtain return of spontaneous circulation. Autopsy imaging indicated residual pericardiac hematoma ( Fig. 4 ). Based on his features, he was suspected of having Loeys–Dietz or Marfan syndrome. We postulated that connective tissue disease had induced Type A aortic dissection with subsequent rupture that had resulted in cardiac tamponade and cardiac arrest. Permission to perform a genetic analysis was not obtained from his parents.

**Fig. 1:**
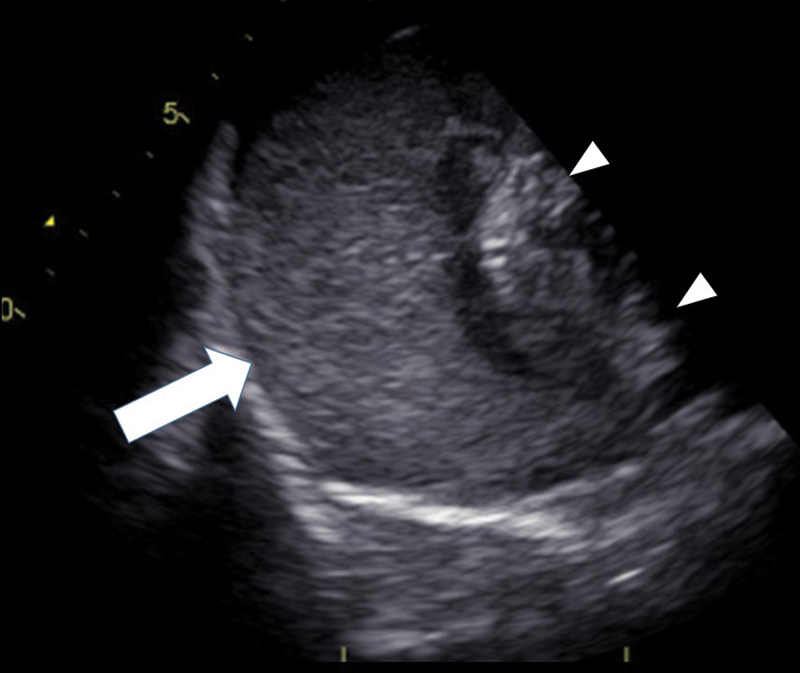
The ultrasound study revealed a high-echoic layer (arrow) surrounding the heart (arrowheads) suggesting clotting cardiac tamponade.

**Fig. 2:**
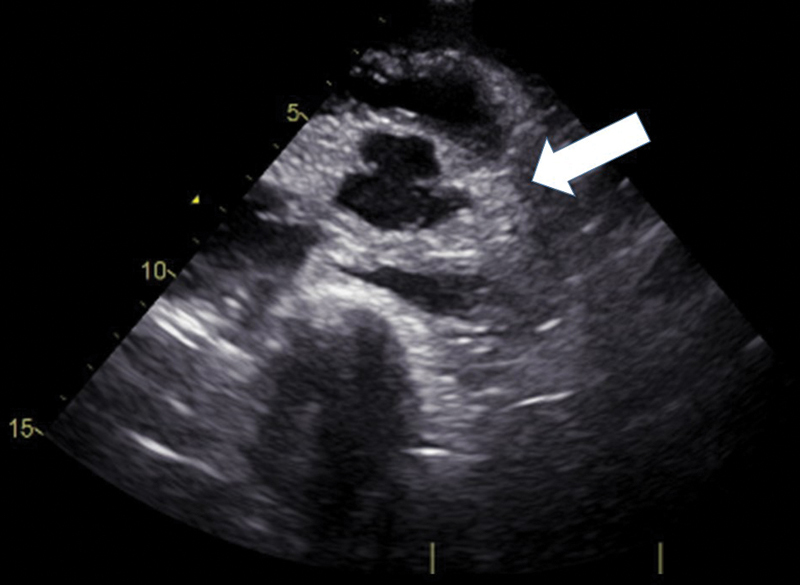
The ultrasound study revealed a high-echoic layer (arrow) surrounding the ascending aorta, suggesting aortic dissection.

**Fig. 3:**
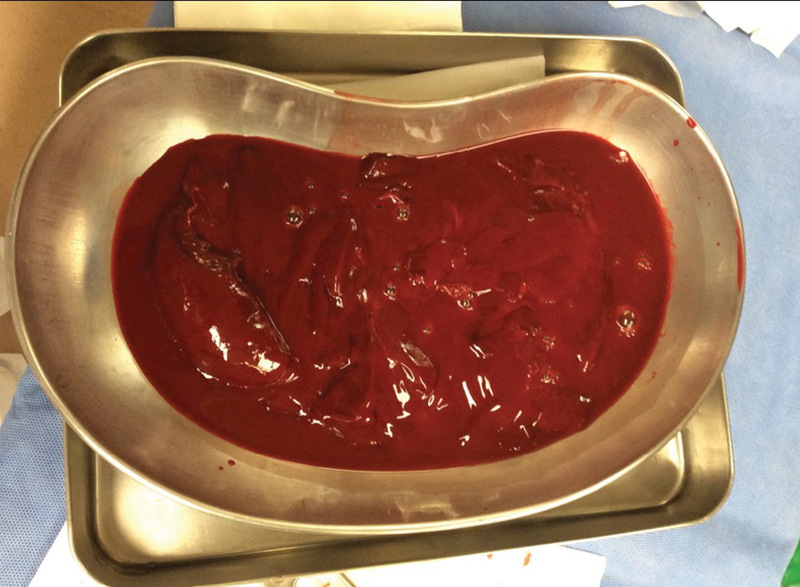
Urgent thoracotomy with pericardiotomy revealed a clot weighing approximately 1 kg.

**Fig. 4:**
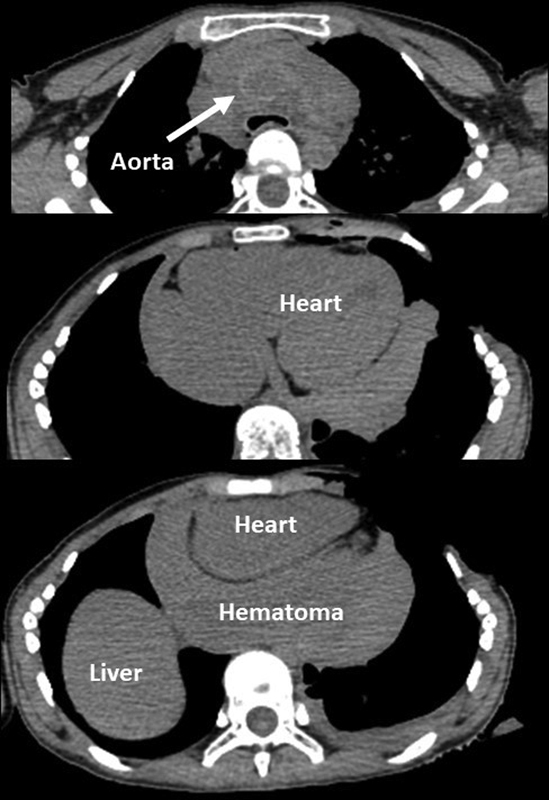
Autopsy imaging. Computed tomography indicated residual pericardiac hematoma.

In cases of hemorrhaging into a closed space, the blood forms a blood clot to achieve hemostasis. During this process, the whole blood separates into a blood clot and serum. When the serum accumulates in one space, it may be detected as fluid by ultrasound. However, it takes some time for the serum to accumulate. Accordingly, a focused assessment with sonography in trauma, which focuses on low-echoic areas to detect serum, is well known to have a high false-negative rate for the acute hemorrhaging state in traumatized patients. 1 Clots are scanned as high-echoic areas on ultrasound studies. If physicians focus not only on low-echoic but also high-echoic areas, the sensitivity for detecting clinically significant blood clots in the human body may be improved.

Unfortunately, the present patient failed to obtain a favorable outcome; however, the urgent resolution of cardiac tamponade by clotting and subsequent treatments might result in a favorable outcome in other patients. 2 3
